# Autophagy as a Therapeutic Target to Enhance Aged Muscle Regeneration

**DOI:** 10.3390/cells8020183

**Published:** 2019-02-20

**Authors:** David E. Lee, Akshay Bareja, David B. Bartlett, James P. White

**Affiliations:** 1 Duke Molecular Physiology Institute, Duke University School of Medicine, Durham, NC 27701, USA; david.e.lee@duke.edu (D.E.L.); Akshay.bareja@duke.edu (A.B.); david.bartlett@duke.edu (D.B.B.); 2 Division of Medical Oncology, Department of Medicine, Duke University School of Medicine, Durham, NC 27701, USA; 3 Duke Center for the Study of Aging and Human Development, Duke University School of Medicine, Durham, NC 27701, USA; 4 Division of Hematology, Department of Medicine, Duke University School of Medicine, Durham, NC 27701, USA

**Keywords:** muscle regeneration, aging, stem cell, immune, macrophage, senescence, exercise, caloric restriction

## Abstract

Skeletal muscle has remarkable regenerative capacity, relying on precise coordination between resident muscle stem cells (satellite cells) and the immune system. The age-related decline in skeletal muscle regenerative capacity contributes to the onset of sarcopenia, prolonged hospitalization, and loss of autonomy. Although several age-sensitive pathways have been identified, further investigation is needed to define targets of cellular dysfunction. Autophagy, a process of cellular catabolism, is emerging as a key regulator of muscle regeneration affecting stem cell, immune cell, and myofiber function. Muscle stem cell senescence is associated with a suppression of autophagy during key phases of the regenerative program. Macrophages, a key immune cell involved in muscle repair, also rely on autophagy to aid in tissue repair. This review will focus on the role of autophagy in various aspects of the regenerative program, including adult skeletal muscle stem cells, monocytes/macrophages, and corresponding age-associated dysfunction. Furthermore, we will highlight rejuvenation strategies that alter autophagy to improve muscle regenerative function.

## 1. Introduction

The physiological process of aging consists of diverse cellular changes including proliferation, metabolism, inflammation, and apoptosis. To identify and provide new therapeutic targets and approaches to counteract the physiological decline of advanced age, a thorough understanding of cellular processes involved is paramount. Autophagy has emerged over the years as an important regulator of age-related changes in various tissues including skeletal muscle. Aging is directly associated with adverse changes to skeletal muscle including a decline in functional capacity which contributes to lower quality of life, reduced independence, and greater mortality in the elderly. The capacity of skeletal muscle to regenerate following injury is, likewise, negatively affected by aging and appears to be associated with alterations in cellular autophagy [1,2]. The regenerative properties of muscle are dictated by the efficient removal and clearance of damaged tissue and the myogenic ability of resident, adult muscle stem cells to replace mature myofibers. Not only does regeneration require autophagy to resolve properly, but in leveraging autophagy, it appears possible to attenuate the age-related decline in skeletal muscle regenerative function.

## 2. Molecular Process of Autophagy

Macroautophagy (hereafter referred to as autophagy and distinct from micro- and chaperone-mediated autophagy as reviewed in [3]) was originally characterized as the apparent self-consumption of mitochondria by the lysosome in mouse kidney cells [4]. Currently, autophagy is understood as a broad process that regulates the delivery of a wide range of proteins and organelles into the lysosome for catabolic degradation. Under pathophysiological conditions, autophagy dysfunction is generally characterized by its insufficiency to remove damaged organelles or debris. Autophagy activation was originally identified as a response to nutrient deprivation in eukaryotic systems [5,6]. We now know that autophagy is activated during various cellular stressors including exercise, endoplasmic reticulum stress, infection, and hypoxia [7]. A functional role of autophagy is expanding to include energy production, cellular remodeling/differentiation and influence over apoptosis [8,9,10,11,12]. Numerous preclinical and clinical efforts target autophagy to treat pathologies such as cardiovascular disease, cancer, and neurodegeneration among others [13]. The process is regulated by a highly-conserved family of proteins referred to as autophagy-related genes (*Atgs*). *Atgs* facilitate specific stages in the autophagic process such as initiation, formation, elongation, and fusion [14].

**Figure 1:**
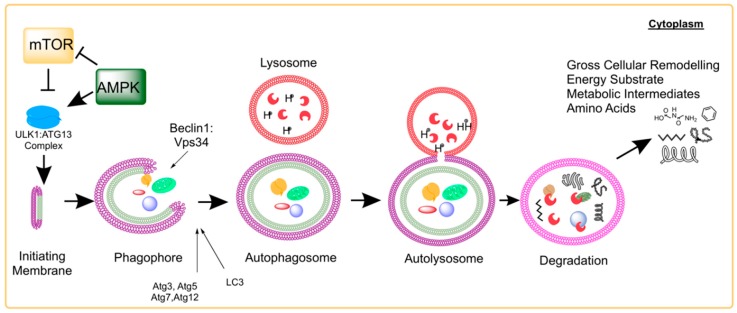
Molecular Events of Autophagy and related Signaling Pathways. Autophagy is a highly-conserved cellular process across eukaryotes from yeast to human. The initiation membrane matures and develops into a phagophore around cytoplasmic compartments containing a variety of macromolecules, organelles, and other cytoplasmic contents. Once fully enclosed, the autophagosome will fuse with the lysosome exposing the contents of the autophagosome to an acidic pH and various digestive enzymes of the lysosome. Following degradation of the contents of the autolysosome, the resulting molecules become available for cytoplasmic utilization (including amino acids, carbon fuel substrates, nucleotides, and reducing cofactors). This process simultaneously allows the cell to undergo drastic and rapid remodeling. Previous research has specifically shown the interaction of mTOR and AMPK in the initial steps of the autophagy process through phosphorylation interaction with the ULK1:Atg13:FIP200 complex.

A number of molecular signals are involved in the coordination and control of the process (readers are referred to a previous review [14]). Transcriptional regulation of autophagy includes the transcription factors JNK [15], NFKappaB [16], HIF-1 [17], and FOXOs [18]. Of key interest is autophagy regulation by the mTOR complexes (mTORC1 and mTORC2) [19]. At high nutrient concentrations, mTOR phosphorylates and inactivates UNC-51-like kinase 1 (ULK1) and Atg13 to prevent the initiation of autophagosome formation [20]. Under starvation conditions, or when autophagy is favored for proteostatic maintenance [21], mTOR dissociation allows the formation of the ULK1:Atg13:FIP200 to initiate autophagy [20]. Subsequent formation and maturation of autophagosomes involves the Beclin1:Vps34 complex, which is negatively regulated by interactions involving the apoptosis promoting members of the Bcl-2 family of proteins [15]. Elongation of the autophagosome membrane utilizes Atg5:Atg12 conjugation and the conversion of cytosolic LC3 (LC3-I) into a membrane-associated PE-conjugated LC3 (LC3-II) facing the interior and exterior of the autophagosome [22]. A summary of the molecular events involved in the autophagy process is illustrated in Figure 1. Once the autophagosome fuses with the lysosome, the contents are broken down into constituent macromolecular precursors that can be reused as raw “bio” material or, alternatively, metabolized. Biochemical markers suggesting this process has been resolved include protein expression patterns of LC3 isoforms and the autophagosome targeting molecule p62. 

Aging is a complex process associated with diminished ability for tissues to maintain biological homeostasis. This is especially relevant in tissues that exhibit age-related changes in autophagic function. In numerous cell types tested, autophagy upregulation is capable of mitigating aging-induced apoptosis and necrosis [23]. Proliferating cells (including stem cells) tend to utilize autophagy for metabolite generation, improved genomic stability and limit oncogenic transformations while postmitotic cells (such as myocytes) rely on autophagy to remove dysfunctional or mutated mitochondria and protein aggregates formed over time [23]. Even in simple eukaryotic models such as *Saccharomyces cerevisiae*, screens for short-lived mutants include various autophagy loss-of-function *Atg* mutants [24]. Similar findings were extended to Unc-51 mutant *Ceanorhabditis elegans* [25] and Beclin mutant *Drosophila melanogaster* [26]. In humans, autophagy downregulation is coincident with numerous pathologies associated with advanced age. Chronic diseases often display reductions in autophagy as demonstrated in brain tissue [27], circulating mononuclear cells [28], connective tissue [29], and cardiac muscle [30]. Wound repair is another relatively unexplored area where age-related changes in autophagy may play an important role [31]. Taken together, these lines of evidence show how autophagy is intricately related to biological aging and senescence.

## 3. Autophagy Effects on Skeletal Muscle Homeostasis, Regeneration, and Aging

Skeletal muscle is a dynamic tissue that is constantly adapting and changing to physical and metabolic demands. As such, autophagy seems to be a key step in healthy muscle homeostasis and physiology [32]. Pathophysiological conditions of muscle that implicate maladaptive autophagy including Duchenne’s muscular dystrophy [33], type II diabetes mellitus/insulin resistance [34], sarcopenia [35], cancer-induced wasting [36], and myotube regeneration [37]; however, the origin of signals inducing autophagy for each scenario seems to differ. In the case of sarcopenia and cachexia, autophagy seems to be an outcome of systemic inflammation signals and dietary hypophagia that contributes to a skeletal muscle phenotype [35,36,38,39]. In other instances, cellular remodeling or tissue repair is seen in dystrophic muscle, and autophagy helps to clear damaged cellular components including myofibrillar components and sequestered cytoplasm [33,37]. These claims are supported when muscle-specific Atg7 knockout in mice resulted in abnormal mitochondrial morphology, oxidative stress, and sarcomeric alterations [32]. Moreover, high-fat diets alter vesicle membrane composition which can also impact rates of autophagy implicating obesity as a modulator of muscle autophagy [40].

In addition to maintaining muscle homeostasis, the activation of autophagy becomes an important factor for skeletal muscle remodeling after stimuli like contractile activity/exercise [41,42]. Not only is autophagy required for skeletal muscle adaptations to physical activity, but autophagy also contributes to improvements in whole body insulin sensitivity [42]. Conversely, mice that maintain basal autophagy rates but lack BCL2-inducible autophagy do not show insulin resistance or alterations in muscle size compared to littermates that are able to activate autophagy above basal levels [34]. The molecular signals responsible include the BCL2-dependent upregulation of Beclin1 as well as AMPK phosphorylation of ULK1 [34,41]. These studies help to define an important role for autophagy signaling in skeletal muscle metabolism and chronic diseases; however, less is known about the function autophagy plays in regeneration. 

Skeletal muscle has extensive regenerative capabilities due to resident stem cell functions and a highly coordinated interaction with hematopoietic/immune cells during the repair process. This complex, yet efficient process results in complete return of morphology and function in healthy muscle. Blocking autophagy by genetic (Atg5, Beclin1) or pharmacological (chloroquine) methods causes accumulation of sarcomeric and nuclear debris following muscle damage in zebrafish showing the key role autophagy plays in the repair process [37]. In the mouse, treatment with a pharmacological inhibitor of global autophagy such as 3-methyladenine (3-MA) after injury slows functional recovery 14 days postinjury [43]. Similarly, mice treated with hydroxy chloroquine (CQ), another chemical inhibitor of autophagy, perturbs muscle regeneration and exacerbates the pathology of *mdx* mice [44]. Genetic inhibition of autophagy through whole body knockdown of Atg16 results in impaired regeneration following cardiotoxin-induced injury [45]. While the importance of autophagy in the regenerative programs have been shown largely using whole body blockage of autophagy, limited investigation has focused on myofiber-specific manipulation of autophagy. Knockout of ULK1 under a myogenin promoter (muscle cell specific) in mice prevents the initiation of autophagy, which impairs the recovery of force production following both cardiotoxin and ischemia/reperfusion-induced damage [46]. These data suggest a possible role for autophagy to clear damaged proteins and organelles to allow myofiber regeneration. Moreover, work in C2C12 cells show autophagy is required for successful differentiation of C2C12 myoblasts, in part by protecting differentiating myoblasts from apoptosis [47]. Together, autophagy may regulate various processes in the regenerating myofiber including proteostasis and survival mechanisms. 

Successful muscle regeneration requires a coordinated effort from various other cell types including mast cells, neutrophils, T regulatory cells, eosinophils, and CD8+ T cells [48]. While each of these cell types contributes specifically to muscle recovery, two cell types that have been studied extensively related to autophagy mechanisms are the muscle satellite cell and the macrophage. The following sections will discuss how autophagy regulates their function and direct or theoretical contributions to muscle regeneration.

## 4. Muscle Satellite Cells

**Figure 2:**
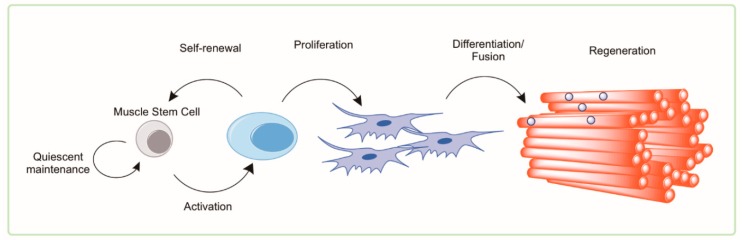
Activation of the Muscle Stem Cell. Adult, resident skeletal muscle stem cells—termed satellite cells—were originally identified in the hind limb muscle of frogs and rats by electron microscopy by 1961 [55]. These cells lie in a quiescent state; dormant with low metabolic flux and little cytoplasm (low energy demand/low energy storage). Following damage by mechanical or chemical stimuli, an activated satellite cell will hypertrophy to accommodate cytosolic machinery and organelle requirements for proliferation. This key functional step leaves the (recently quiescent and still relatively small) satellite cell with little in the way of energy stores or metabolic machinery (i.e., mitochondria) but an extremely high demand for energy substrates, metabolic intermediates, and amino acids. This state of high energy demand/low energy storage in accompanied by a simultaneous remodeling of the cell architecture. By using autophagy as a process to remove aged, unnecessary, or damaged components, the young/healthy activated satellite cell is capable of recycling those components into the building blocks needed to further proliferate. The proliferated satellite cells will asymmetrically divide allowing the self-renewal of the activated stem cell into the quiescent state; likewise requiring significant cytoplasmic reorganization and remodeling. The satellite cell progeny will undergo subsequent differentiation into mature myogenic cells that are able to fuse with the damaged myofibers accomplishing successful and complete regeneration.

Adult skeletal muscle maintains a relatively remarkable ability to regenerate and recover following mild damage or insult throughout much of the lifespan [49]. The functional adult stem cell of skeletal muscle is the satellite cell [50]. Termed this way based on its appearance at the periphery of muscle fibers, the satellite cell is the source of replenishment of myonuclei following damage and is called upon to meet the transient demands of myofiber hypertrophy and repair [51,52,53,54]. Quiescent satellite cells are identified as Pax7^+^/Myod^−^ cells that are capable of asymmetric cell division and forming mature myonuclei when transplanted into injured muscle. Successful regeneration requires two stages of satellite cell function: proliferation and fusion/myogenesis. Both phases of repair show evidence of dysfunction with advanced age. The function of satellite cells is measured by their ability to activate from quiescence and undergo asymmetric proliferation into (1) daughter cells that are capable of further proliferation and differentiation into mature muscle fibers and (2) daughter cells that are capable of reentering quiescence. Therefore, we refer to satellite cell function as their ability to proliferate and/or undergo myogenesis. A summary of the general mechanism of myogenesis and myofibrillar regeneration is shown in Figure 2.

### 4.1. Satellite Cells in Health and Aging 

Aging has long been known to be inversely related to the proliferative potential of satellite cells [56,57,58]. Depletion of satellite cells drastically inhibits proper muscle regeneration in response to damaging stimuli in old and young mice [59]. Early investigation used eccentric contraction-induced injury in animals combined with irradiation (a method to prevent satellite cell proliferation) to demonstrate a contribution of satellite cells to hypertrophy and recovery. In irradiated mice, isometric torque recovery was reduced by ~25% in mice that were irradiated and damaged compared to nonirradiated controls [60]. Similarly, stretch-induced damage was able to upregulate key myogenic regulatory factors, including Mrf4, MyoD, and myogenein in quail muscle; however, myogenin gene expression was reduced if the muscle had been irradiated prior to stretch overload [61]. These studies suggest that satellite cells contribute to recovery from physiological forms of injury such as contraction and overload, which are translatable to injuries observed in the elderly or sarcopenic [62]. Conditional ablation of *Pax7*-expressing satellite cells suggests a minimal role in the development of sarcopenia (under sedentary conditions) as measured by specific force and muscle morphology comparing young (~5 months) and aged (~22 months) mice [63]. Similarly, the *Pax7*-dependent contribution of BrdU to myofiber nuclei was also shown to be minimal in unchallenged muscle [63]. While these studies demonstrate a role for *Pax7* positive satellite cells in regeneration, several groups have reported age-associated decrements in the number of satellite cells [64,65,66]. Not only is the number of satellite cells reduced in aged muscle but the ability of the cell to exit quiescence and activate is negatively impacted [67]. The mechanism(s) directing the age-related impairments in satellite cell function are uncertain; however, there is evidence for systemic, intercellular, and niche-associated contributors [68,69,70]. 

Satellite cells isolated from aged mice can be transplanted into injured muscles of young recipients and contribute to regeneration but fail to repair myofibers and replenish the quiescent resident stem cell pool to a similar extent of young donor cells [2,71]. This model has repeatedly shown the limited ability of satellite cells from aged donors to expand, self-renew, and fuse into myofibers regardless of host age supporting some intrinsic change in the satellite cell that cannot be reversed by engraftment into a youthful environment [71,72]. Several reports have elucidated cellular mechanisms responsible for age-associated dysfunction which include p38α/β mitogen-activated protein kinase (MAPK) signaling axis, FGFR-Sprouty1 signaling axis, JAK2-STAT3 signaling axis, and p16^Ink4a^ [56,67,70,71]. Recently published evidence further highlights a function for the tumor suppressor gene p53 in regulating cell cycle progression in activated muscle stem cells which is decreased in aged mice and leads to reduced proliferative and regenerative function [73]. Alterations in the satellite cell niche of older mice alters Notch signals [57] which, in turn, upregulates Mdm2 and limits p53 protein. This results in a propensity for impaired satellite cell proliferation and regenerative decline [73]. Age-related intrinsic changes can activate and/or repress many of the pathways mentioned, resulting in a limited ability to self-renew, and successfully differentiate into mature myofibers.

### 4.2. Autophagy and Muscle Satellite Cells

**Figure 3:**
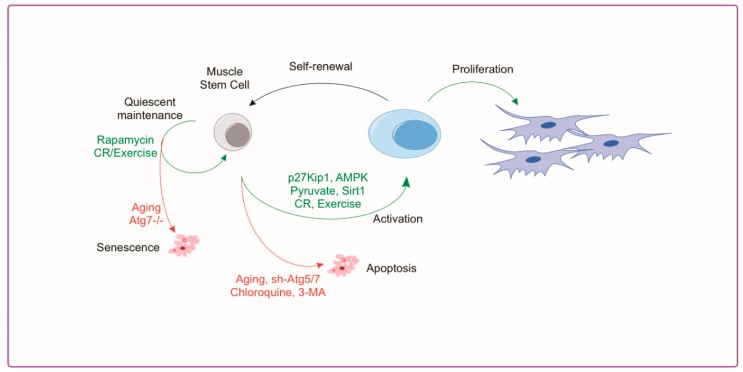
Aging and Autophagy Directly Impact Muscle Stem Cell Activation. Autophagy in the muscle stem cell can directly affect cell fate decisions including the maintenance of quiescence or entering into senescence. These can be controlled by aging, exercise, and caloric restriction or by direct manipulators of autophagy such as rapamycin and genetic ablation of the Atg system. Similarly, muscle stem cell activation requires sufficient energy substrate that can be limiting when autophagy is reduced (i.e., aging or with the autophagy inhibitiors chloroquine or 3-MA). Restoring the energetic signaling through AMPK activation (AICAR) or by supplementing the energy stores through pyruvate can help to replenish stem cell activation.

The importance of skeletal muscle autophagy is evident as impairments in autophagy in muscle tissue cause myopathy [32], altered regeneration [46], and accelerated aging [74]. The role of autophagy in satellite cell biology is less clear but several studies have helped to elucidate a better understanding in this area. Reports from our group [2] and others [1,75] have demonstrated that autophagy plays a crucial regulatory role in satellite cell quiescence, activation, differentiation and apoptosis. Advanced aged demonstrates a clear reduction in autophagy markers in isolated MuSCs [1,2]. Tang and Rando [76] show that autophagy is critical for activation and proliferation by acting as a temporary energy source to fuel initiation of proliferation. This is necessary because the relatively sparse cytoplasm and mitochondria of the satellite cell provide meager energy substrate when exiting quiescence; therefore, relying on autophagy to produce new biomass [77]. Conversely, aging muscle stem cells have a reduction in autophagy which, when rescued, has a rejuvenating effect and suppresses senescence [1]. In addition, impairment of autophagy in young satellite cells leads to a reduction in proliferation and myogenesis similar to what is observed in aged cells [1]. Aged satellite cells are inherently susceptible to apoptosis following cellular stress compared to healthy cells demonstrating a shift from autophagy to apoptosis with aging [78]. Recently published work by our group shows advanced age drives muscle stem cell dysfunction, in part through impairments in the AMPK/p27^Kip1^ pathway [2]. MuSC activation requires the induction of AMPK/p27^Kip1^ signaling to promote autophagy and subsequent proliferative/myogenic function. Aged MuSCs had suppressed AMPK/p27^Kip1^ signaling associated with reductions in autophagy and proliferation with increased apoptosis and senescence. Genetic or pharmacological activation of AMPK or p27^Kip1^ was effective in inducing autophagy and returning myogenic potential of aged MuSCs in addition to blocking the induction of senescence markers [2]. A summary of autophagy associated mechanisms in the satellite cell and approaches used to manipulate autophagy are shown in Figure 3. Although these studies suggest a key role for autophagy in satellite cell activation and regeneration, further investigation is warranted. Specifically, if autophagy is required to meet the energetic demands of satellite cell activation, then which autophagy-derived substrates are necessary for successful activation and how are these substrate concentrations affected in aged MuSCs? Furthermore, does the age-related reduction in MuSC autophagy contribute to the loss in satellite cell number with age?

## 5. Monocytes and Macrophages

Alongside satellite cells, the innate immune system, specifically myeloid cells, plays a principal role in development, homeostasis, and regeneration of skeletal muscle [79,80,81,82]. Following damage, muscle exhibits traditional inflammatory responses including rapid and dramatic infiltration of monocytes and macrophages. Monocytes are derived from a hematopoietic stem cell (HSC) lineage and are activated and recruited to tissues where they differentiate into macrophages [83]. Recently, the importance of monocytes throughout the muscle repair process has become clear from early migration and removal of damaged tissue, inflammatory vs. anti-inflammatory macrophage roles, and physiological impacts of aging on regenerative potential [84,85]. Monocytes are key intermediaries between the innate and adaptive immunity and the role of autophagy is pertinent to all functions of the monocyte. Monocytes are critical in directing immune responses and the repair and maintenance of tissues throughout the body. This is done by chemotaxis towards inflammation such as tissue damage and infection where they can aid and orchestrate the repair process. Monocytes are a short-lived (t_1/2_ = 3 days) and highly mobile cell residing in the blood and spleen which respond to inflammation by differentiation into macrophages and dendritic cells as well as tissue specific cells such as the Kupffer cells of the liver. The process of differentiating from a monocyte to a macrophage dictates the extent and duration of both immune responses and tissue remodeling. Without appropriate stimulation to differentiate, monocytes undergo apoptotic cell death and are cleared from the system. Here we will focus on the role autophagy plays on monocyte differentiation with age and the impact this has on macrophage function during regeneration. In order to fully appreciate the regenerative function of these immune cells, one must appreciate the phenotypic spectrum and heterogeneity of the macrophage population recruited during the regenerative process.

### 5.1. Autophagy in Monocyte Development and Macrophage Differentiation

Monocytes are derived from quiescent HSCs found in the hypoxic bone marrow environment. This environment limits oxidative metabolism and nutrient availability to HSCs which are typical activators of the autophagic process [86,87,88]. In fact, evidence shows that basal autophagy is elevated in human HSCs [89]. The importance of autophagy in quiescent of HSCs is partly due to a greater need for catabolism to remove damaged cellular components/organelles that are not actively removed by cell division [90]. Others have shown the value of autophagy as a source for ATP and to mitigate production of ROS through mitochondrial clearance by mitophagy [91,92]. FOXO3A and Atg7 are specific mechanistic pathways that have each been shown to contribute to quiescent maintenance through autophagy in HSCs [93,94]. Furthermore, evidence implicates an age-related decline in HSC autophagy [95] and function which can be rejuvenated by rapamycin treatment in mice [96]. This is highlighted through use of Tsc1-deficient HSC mice with constitutive activation of mTOR which show upregulated mitochondrial biogenesis, ROS production, and HSC activation that can be reversed by rapamycin treatment [97]. Additionally, Akt activation results in loss of HSC populations and increased cycling leading to leukemia in mice [98]. These studies have helped to establish a key role for autophagy in the maintenance of HSC quiescence as well as activation through an mTOR dependent route. However, the importance of autophagy extends beyond the HSC to the monocyte–macrophage differentiation process.

Monocytes are short-lived in circulation and undergo apoptosis unless stimulated by inflammatory factors such as TNF to upregulate survival pathways. Upon activation by inflammatory signals, monocytes use autophagy as a means to differentiate into macrophages or dendritic cells [99]. The specific signals stimulating the monocyte–macrophage transition have been shown to require autophagy in an ULK1- and Atg7-dependent manner in human and mouse primary cells [100,101]. Using both pharmacological inhibition of Atg7-/- mouse cells, monocytes were unable to differentiate when stimulated by CSF1 [100]. CSF2 stimulation of monocyte differentiation also appears to depend on autophagy through a Beclin1/Bcl2 interaction mechanism [99]. In addition to monocytes triggering towards a macrophage fate, autophagy can impact the polarization of the subsequent cell.

### 5.2. Monocyte and Macrophage Heterogeneity

**Figure 4:**
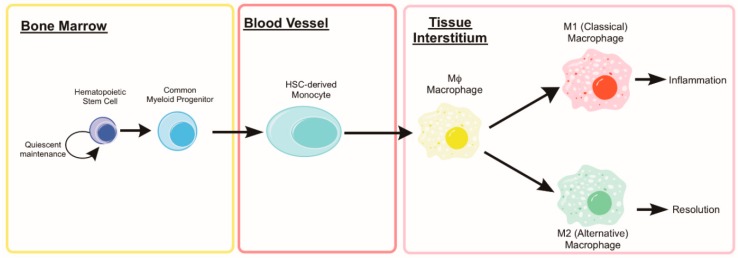
Development of Bone Marrow-derived Macrophages. Hematopoietic stem cells (HSCs) reside in their hypoxic niche within the bone marrow and maintain a quiescent state. When activated, the HSC can generate daughter cells of the common myeloid progenitor lineage and subsequently, into circulating monocytes. When stimulated by inflammatory cytokines (i.e., IL-6 or TNF, such as following muscle damage), monocytes undergo transcriptional changes that prevent apoptosis and enhance autophagy machinery during the differentiation into a macrophage. Mature macrophages (both classical and alterative) rely heavily on autophagy to aid in their function of clearing the damaged tissue and cellular debris that is the result of muscle injury.

During the early stages of the recovery process (roughly 0–48 h post injury), neutrophils help to condition the tissue environment at the site of injury helping to direct subsequent regenerative immune events. Monocyte extravasation in to the muscle is controlled by chemotaxis towards pro-inflammatory cytokines such as interferon-γ (IFNγ) and tumor necrosis factor (TNF). Following classical activation and recruitment to the damaged muscle, monocyte differentiation will lean towards a pro-inflammatory macrophage (M1) designed to ingest cellular debris and necrotic tissue to combat the initial insult and direct the subsequent immune response. Following the initial M1 response, monocytes will begin to differentiate towards alternatively activated, anti-inflammatory macrophages (M2), which reduces local inflammation and tampers the immune response to promote tissue remodeling (see Figure 4). The characterization of macrophages as M1 versus M2 phenotypes is an approach which fails to account for the diverse continuum of macrophage phenotypes [102]. In some scenarios, macrophages isolated from damaged muscle can express transcripts associated with either/both M1 and M2 phenotypes and the change in expression of these transcripts can be highly transient [103,104,105]. NF-κB transcriptional control of selective autophagy appears to be responsible for some aspects of M2 polarization [106]. Through inhibition of autophagy, NF-κB activity can force M2-polarized macrophages to secrete relevant levels of M1-cytokines [107]. Others have found that autophagy activation through rapamycin treatment leads to the classical M1 phenotype in macrophages from human blood [108]. These studies suggest that greater autophagy activation is directly related to the differential polarization of macrophages in response to stimuli [109]; however, a knowledge gap still exists in the field supporting autophagy as a direct contributor to either macrophage phenotype. Many of these studies rely on transcriptional control of NF-κB on autophagy or pharmaceutical modulators of autophagy each of which could have many confounding effects in the cell. Further evidence is needed to solidify a causative relationship between cellular autophagy and remodeling in macrophages, especially in the context of muscle regeneration and aging. 

### 5.3. Autophagy and Macrophage Function and Phenotype

It is clear that autophagy is a critical component to the efficient function of immune cells to clear pathogens or cellular debris. This is highlighted by macrophage function during skeletal muscle repair [110,111]. We refer to macrophage function as the ability of the cell to ingest and eliminate debris from a site of injury while aiding in the coordination of inflammation/resolution through cytokine production and excretion. In mature macrophages lacking ATG7, fewer autophagosomes are formed; there is a reduction in functional surface receptors, increased glycolytic flux, and greater basal inflammation [112]. ATG7 is essential to the elongation of the phagosomal membrane and lysosome function so this alters the inherent function of macrophages to use lysosomal means to degrade phagocytosed cellular debris. This implies macrophage autophagy is needed to clear away damaged muscle tissue and when this is prolonged, inflammatory cytokine production will persist. Others have used similar *Atg*-deficient mouse models to show that there is a need for effective autophagy to promote an anti-inflammatory environment through LC3-associated phagocytosis and appropriate cytokine signal release [113,114]. 

In addition to regulating macrophage function, autophagy appears to play a role in phenotype as well. By using a LysM-Cre recombinase system with *Atg7^fl/fl^* mice, macrophage-specific autophagy was shown to play a role in M1/M2 polarization potential. When differentiated macrophages are unable to use autophagy, the macrophage population shifts towards a proinflammatory M1 phenotype along with greater glycolytic metabolism [115]. Conversely, the proportion of M2 macrophages was reduced by limiting fatty acid oxidation through lysosomal acid lipase—a portion of the autophagic degradation system [116]. The interaction between autophagy and macrophage polarization is unsurprising because of the initial ULK1 signal in monocytes initiating the differentiation response. ULK1 directly interacts with mTOR and autophagy is inhibited when nutrient stimulation of mTOR is high [117]. When mTOR is constitutively active, IL-4-stimulated macrophage polarization is tilted towards an inflammatory M1 phenotype [118,119]. 

### 5.4. Age-Associated Suppression of Monocyte/Macrophage Function

Immune cells are subjected to an age-associated decline in effective response, termed immunesenescence [120]. Although all aspects of immunity are affected by age, the impact to the innate system is particularly significant [121]. Aging of the innate immune system is accompanied by an increased number of circulating monocytes which have reduced effector functions (i.e., chemotaxis, phagocytosis, and antigen presentation) [121]. Evidence demonstrates age-related deterioration of NO burst, fewer toll-like receptor antigen recognition particles, and greater inflammatory cytokine release by macrophages [122,123,124,125]. Accompanying this is an increased level of chronic systemic inflammation that is in part associated with innate immunesenescence [126]. The complex mechanisms responsible for immunesenescence are unclear but the reduced immune effector function and increased low level inflammation contribute to reduced tissue homeostasis and repair after damaging insult. These deficiencies of innate immunity can result in age-related frailty and greater mortality for the elderly [127,128]. Interestingly, many of the key roles for autophagy in macrophages become dysregulated with advanced age unveiling a key link between aging and macrophage function [127,128,129].

**Figure 5:**
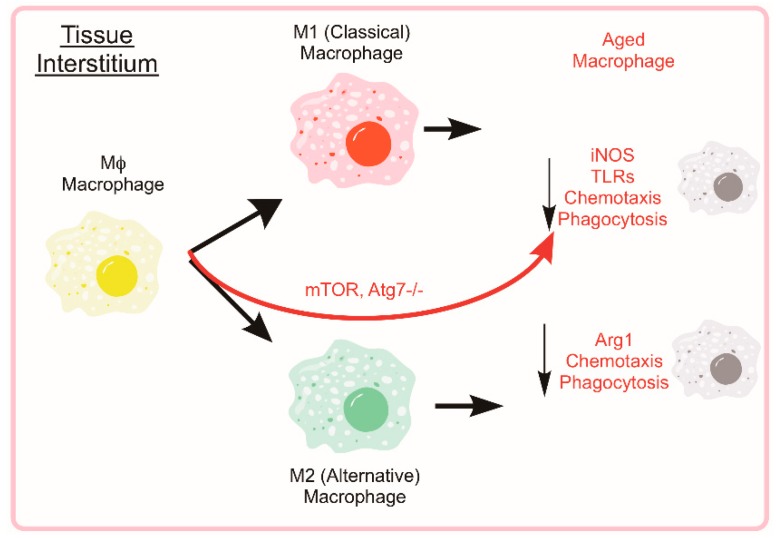
Aging Negatively Effects Macrophage Autophagy and Associated Phenotype/Function. The mature macrophage uses autophagy as part of the cell’s inherent function to phagocytose and eliminate pathogens or debris. The aged macrophages tend to have impairments in the autophagic functions, phagocytosis, and chemotaxis. While aged individuals tend to have elevated circulating inflammatory markers, aged M1 and M2 macrophages show reductions in iNOS and Arg1, respectively. By limiting autophagy through Atg7 genetic ablation or constitutive mTOR activation, M2 macrophages show a shift towards M1 cytokine secretion.

Evidence supports age-associated changes in macrophage function across the lifespan. Early studies showed altered response and expression of toll-like receptors in aged mice compared to young [123]. Monocytes isolated from young and old mice that are then stimulated ex vivo with LPS show an age-related reduction in the secretion of pro-inflammatory (M1) cytokines including IL-6, IL-1β, and TNF-α [130]. Similarly, when stimulated with IFN-γ and IL-4, aged monocytes expressed the markers Arg1, Ym1, and FIZZ1 to a lesser extent than young counterparts [130]. This series of experiments indicates that macrophages from old mice (>2 years) produce fewer cytokines than young mice both under situations of pro-inflammatory and anti-inflammatory stimuli. Moreover, some investigations into human inflammatory markers have shown an imbalance in the circulating IFNγ- and IL-4-related markers in elderly humans (81–100 years) compared to young [131]. Age-associated changes to macrophage function and related effects of autophagy manipulation are summarized in Figure 5.

## 6. Rejuvenation of Autophagy for Muscle Regeneration

After having established the importance of autophagy in various aspects of skeletal muscle repair, autophagy, and related pathways present an obvious therapeutic target to enhance muscle function and regenerative capacity. Exercise and caloric restriction (CR) are two established methods shown to induce autophagy in several tissues [42,132,133,134,135]. In addition, exercise and CR enhance cellular function of both MuSCs and macrophages [136,137,138,139,140]. The following sections will discuss how autophagy may play a role for each approach. 

### 6.1. Rejuvenation of Satellite Cells through Exercise and CR

#### 6.1.1. Exercise

Exercise is an established method to prevent the onset of sarcopenia. In addition, exercise may also aid to enhance regenerative capacity in aged muscle. Moderate intensity treadmill running is capable of increasing the number of MuSCs isolated from hindlimb muscles in young and old mice while increasing aged MuSC proliferative function [64,141]. In regard to signaling, voluntary wheel running upregulated Wnt/β-catenin activity, irrespective of structural damage [142]. Thus, suggesting a possible mechanism to explain why exercise can increase satellite cells numbers of young and old muscle. To the best of our knowledge, the effects of physical activity on satellite cell autophagy have not been investigated in satellite cells/MuSCs. Considering exercise is able to induce autophagy in peripheral tissues that are not actively performing contraction [133], satellite cells may receive some exercise-induced signals to increase autophagy similar to what was observed in nonmuscle tissue [133]. Well-controlled studies directly linking exercise with enhanced satellite cell autophagy are needed. An increase in satellite cell autophagy could help to explain a mechanism for enhanced satellite cell number and function with exercise. 

#### 6.1.2. Caloric Restriction

An additional intervention shown to induce global autophagy and related extension in health/lifespan is caloric restriction [143]. The benefits of longevity through moderate reductions in caloric intake have been shown in numerous models [136,137,143,144]. Moreover, CR has shown beneficial effects on adult stem cell function across various tissues [145]. Even short term (12 weeks) CR improves the number and proliferative function of satellite cells in young mice (eight weeks) with the benefits extending to aged mice (21 months old) [146]. In addition to improved regenerative capacity, CR also enhances engraftment capability of MuSCs isolated from ad libitum fed donors [146]. The contribution of autophagy to the pro-regenerative effects of CR is unclear as yet; however, Cerletti et al. [146] show improvements in oxidative characteristics and mitochondrial markers suggesting altered energy status of the MuSC [147]. Extending upon these results, a similar CR regiment in aged mice resulted in a greater phosphorylation of AMPK and p27^Kip1^ in activating aged MuSCs [2]. These data suggest CR may “prime” the aged MuSC to initiate autophagy upon activation. Further investigation is certainly warranted to understand CR-sensitive pathways in quiescent and activated satellite cells/MuSCs. 

### 6.2. Rejuvenation of Macrophages through Exercise and CR

#### 6.2.1. Exercise

Little has been shown on how exercise training might improve immune function in the elderly or mitigate the immunosenescent phenotype. Early studies have shown a connection between exercise and macrophage phagocytic function in athletes [148]. A 10-week exercise program with varying exercise intensities improves immune function compared to pre-exercise measurements [149]. In addition, exercise increased neutrophil migratory function in old adults [150]. In the mouse, aging reduced the cytokine production and function of both M1 and M2 macrophages; exercise training appears to sensitize macrophages to LPS-induced cytokine production [151]. Long-term treadmill running in mice has been shown to elevate markers on M2 macrophages (higher CD163 and lower TLR4) in high-fat diet-fed mice adipose tissue suggesting an exercise-induced alternative macrophage promoting phenotype. While these data showed a correction of high-fat diet-induced M1 markers by exercise (Cd11c and ICAM-1), exercise training in healthy mice did not have any effect on the macrophage phenotype [152]. To date, no study has investigated the association between immune cell autophagy and exercise. However, autophagy regulates macrophage differentiation and therefore exercise could enhance muscle regeneration by promoting the transition between pro and anti-inflammatory macrophages. 

#### 6.2.2. Caloric Restriction

Similar to the MuSC, macrophage polarization and function seems to be sensitive to caloric restriction [153]. Greater M2 macrophage-related cytokine production and cellular markers were measured in the adipose tissue of caloric restricted mice [153]. It remains unclear if these changes are associated with alterations in cellular autophagy. Caloric restriction has been shown to enhance the anti-inflammatory characteristics of macrophages, but it remains unclear how this might relate to autophagy function in the context of muscle regeneration.

### 6.3. Pharmacological Inducers of Autophagy

**Figure 6:**
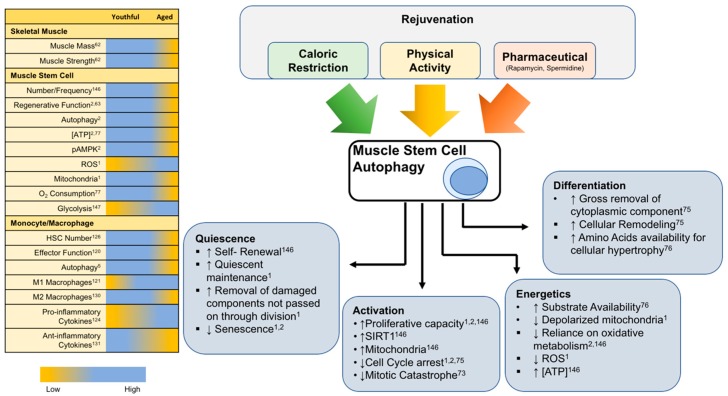
Rejuvenation of Muscle Stem cell autophagy. **Left**: Chart summarizing various physiological and functional parameters involved in skeletal muscle regeneration and how they are susceptible to age-related alterations. **Right**: Conceptualization of the rejuvenation potential targeting stem cell autophagy and how various cellular functions are affected. Caloric restriction, physical activity, and certain pharmaceuticals are known to affect stem cell autophagy. The role of autophagy in the stem cell is multifaceted with implications in quiescence vs. senescence, activation/proliferation vs. apoptosis, and differentiation outcomes. A substantial contribution of autophagy in the function of a muscle stem cell is alterations in the cellular metabolic landscape or energetics.

As has been alluded to, the pharmacological induction of autophagy represents a promising strategy to improve stress resistance and regeneration of skeletal muscle. Spermidine and rapamycin are two examples of drugs that have been studied for their autophagy-inducing effects and lifespan extension in rodent models [154,155]. While rapamycin acts directly on mTOR, spermidine’s polyamine effects on histone acetylation status upregulates various autophagy-related transcripts and suppresses necrosis [156]. The positive benefits of spermidine in muscle tissues of mice and rats have been shown by mitigating age-related muscular atrophy as well as functional myopathies that originate from autophagy failure [157,158]. The autophagy-inducing effects of spermidine and rapamycin have also been detailed specifically within the muscle stem cell [1,159,160]. Satellite cells were isolated from aged mice and treated ex vivo with either rapamycin or spermidine and a dramatic increase in autophagy was measured by immunofluorescence of LC3 puncta. Gracia-Prat et al. further demonstrated that the pharmacological induction of autophagy reverses age-related declines in mitochondrial morphology and regenerative function [1]. Spermidine also modulates macrophage polarization in mice towards reduced inflammation, though some evidence suggests the autophagy inducing effects of rapamycin more directly target T lymphocytes [161,162]. Taken together, these agents act as “caloric restriction mimetics” to induce autophagy and contribute to improvements in lifespan of mice. Specifically, the effects of autophagy induction show promise as it related to therapies targeting muscle stem cell myogenic capacity. Figure 6 illustrates general age-related differences across various components of the regenerative process in addition to known lifespan/healthspan approaches and their common effects to enhance tissue regeneration.

## 7. Conclusions and Future Perspectives

The ability to repair damaged tissue and continuously respond to stressful stimuli is essential to preserve whole body function throughout life. Muscle stem cells and macrophages are an integral part of this process and their loss of function with age contributes to degenerative diseases. This review highlights how muscle stem cells and monocytes/macrophages are essential for skeletal muscle homeostasis and regeneration. A common theme among these cell populations is the idea that autophagy is a key process that is altered in aged cells leading to functional decline. Autophagy is no longer an emerging regulator of cellular function but has consistently been shown to play a central and important role, especially in the context of aging. Stem cells, in particular, show dysfunctional autophagy during initial stages of activation while caloric restriction and physical activity allow a sensitization to autophagy with beneficial outcomes in cellular activation and function. The exact role for autophagy in muscle regeneration will be complex considering the temporal nature and diverse cell types contributing to the regenerative program. However, global induction of autophagy appears beneficial to the regenerative capacity in the aged muscle. Continuing to uncover the molecular events responsible for age-related perturbations in these pathways is critical for exposing pharmaceutical targets to combat the aging process and improve tissue regeneration in aged individuals.
